# Downregulation of IFNG in CD4^+^ T Cells in Lung Cancer through Hypermethylation: A Possible Mechanism of Tumor-Induced Immunosuppression

**DOI:** 10.1371/journal.pone.0079064

**Published:** 2013-11-11

**Authors:** Fang Wang, Jian Xu, Quan Zhu, Xuejun Qin, Yan Cao, Jiangfang Lou, Yuqiao Xu, Xing Ke, Qing Li, Erfu Xie, Lixia Zhang, Ruihong Sun, Liang Chen, Bingliang Fang, Shiyang Pan

**Affiliations:** 1 Department of Laboratory Medicine, The First Affiliated Hospital of Nanjing Medical University, Nanjing, China; 2 National Key Clinical Department of Laboratory Medicine, Nanjing, China; 3 Department of Cardiothoracic Surgery, The First Affiliated Hospital of Nanjing Medical University, Nanjing, China; 4 Department of Thoracic and Cardiovascular Surgery, The University of Texas M. D. Anderson Cancer Center, Houston, Texas, United States of America; University of Navarra, Spain

## Abstract

Tumor survival is significantly correlated with the immune response of patients. IFNG plays an important role in the tumor host response and decreased IFNG expression is often observed in lung cancer. Studies have shown that CpG island hypermethylation plays a critical role in transcriptional silencing of IFNG gene expression. However, there is limited understanding regarding the molecular mechanisms of altered methylation, and whether the tumor microenvironment has any effect on DNA methylation and IFNG production. In the current study, we demonstrate that plasma and intra-cellular IFNG levels are significantly lower in lung cancer patients. Hypermethylation of the IFNG promoter in CD4^+^ T cells and plasma IFNG was negatively correlated. CD4^+^ T cells from healthy individuals co-cultured with SPC-A1 cells generated lower levels of IFNG after activation, elevated expression of DNA methyltransferases (DNMTs), and exhibited hypermethylation of the IFNG promoter. In conclusion, decreased IFNG expression of CD4^+^ T cells co-cultured with lung cancer cell is associated with IFNG promoter hypermethylation. Our study suggests that interaction between lung cancer cells and CD4^+^ T cells induces DNMT expression and IFNG promoter hypermethylation in CD4^+^ T cell, which may serve as an important mechanism of tumor-induced immunosuppression.

## Introduction

Lung cancer has a short 5-year survival rate since it is difficult to diagnose and treat at an early stage [1]. Although the mechanisms of lung cancer initiation are not fully understood, it is believed that the tumor escapes immune surveillance [2].

Cytokines are part of a complex immune response that can assist the development of cancer, as well as eliminate it. There is a close relationship between tumor progression and dysregulation of cytokine expression, as seen for IFNG, TGF-β, and IL-17 [3], [4]. Among these cytokines, IFNG, which was discovered in 1965, has a reputation for helping guard against neoplastic disease. IFNG inhibits proliferation, sensitizes tumor cells to apoptosis, up regulates MHC class I and class II expression, and stimulates antitumor immune activity [5], [6]. Decreased IFNG serum levels have been linked to shorter survival in lung cancer [7]. Therefore, elucidating the molecular mechanisms of IFNG in tumorigenesis is critical to have a more clear understanding of tumor pathogenesis.

Epigenetic changes such as histone modifications, DNA methylation, and variations in chromatin structure have been shown to be important for the selective transcription of cytokine genes in T cell subsets. Among these, DNA methylation has been studied widely in relation to cytokine gene expression [8]–[10]. In this study, we considered the inverse correlation of IFNG expression and DNA methylation in lung patients. More importantly, to evaluate whether lung cancer cells could impact the methylation status of immune cells by down regulating IFNG expression, we established an in vitro transwell culturing system and then investigated CpG methylation of the IFNG promoter in CD4^+^ T cells.

## Results

### IFNG Levels of Healthy Controls and Lung Cancer Patients

**Figure 1:**
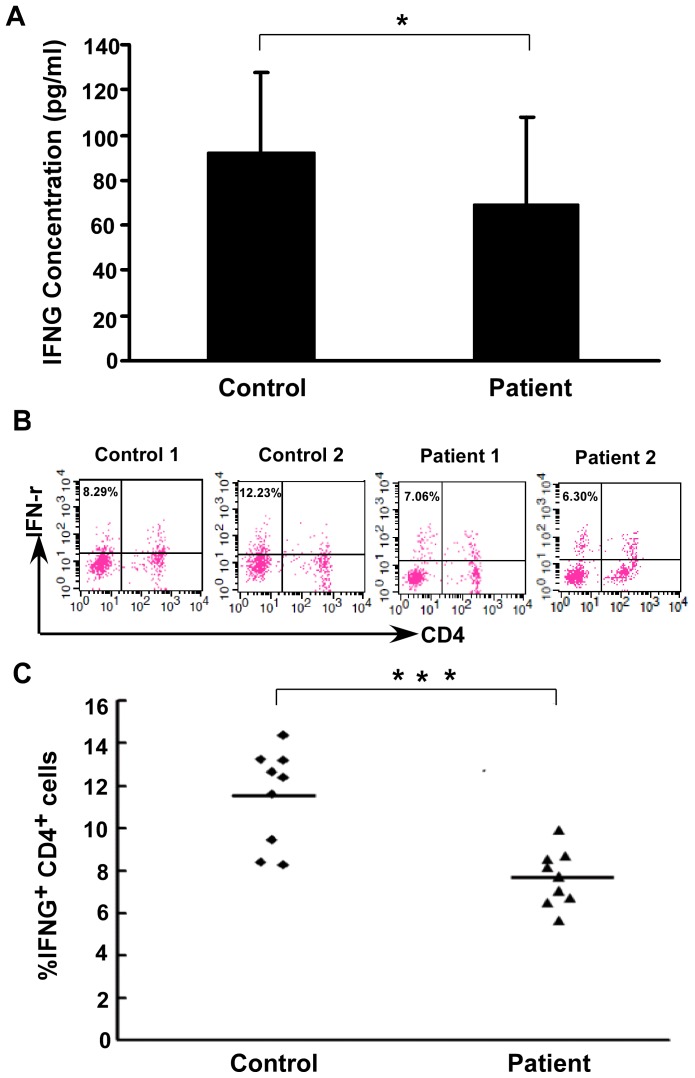
IFNG levels of healthy controls and lung cancer patients. (**A**) Comparison of plasma IFNG levels in study groups. ELISA was used to measure plasma IFNG levels in 30 lung cancer patients and 30 healthy controls. Plasma IFNG levels in patients and healthy controls were 69.30±38.56 pg/ml and 92.62±34.75 pg/ml respectively (*P = 0.017). (**B**) Representative results of IFNG expression by CD4^+^ T cells from lung cancer patients (n = 2) and healthy controls (n = 2). (**C**) Flow cytometric analysis of IFNG levels in study groups. Data are the individual frequency from lung cancer patients (n = 9) and healthy controls (n = 9). Horizontal bars indicate the group means. Independent t tests were used to calculate *P* values (***, *P*<0.001).

ELISA was used to detect plasma IFNG levels (**Fig. 1A**). The IFNG levels in lung cancer patients were significantly lower (69.30±38.56 pg/ml) than in healthy controls (92.62±34.75 pg/ml, *P* = 0.017).

IFNG is produced primarily by CD4^+^ T cells, but also by NK cells and CD8^+^ T cells. To reflect IFNG expression in CD4^+^ T cells, we evaluated CD4^+^ T cells by flow cytometric analysis. PBMCs of 9 lung cancer patients and 9 healthy controls were collected and IFNG were detected. A lower frequency of IFNG producing CD4^+^ T cells were detected in lung cancer patients compared with healthy controls (**Fig. 1B–C**, P<0.001).

### IFNG Gene Promoter Methylation is Negatively Correlated with IFNG Levels

**Figure 2:**
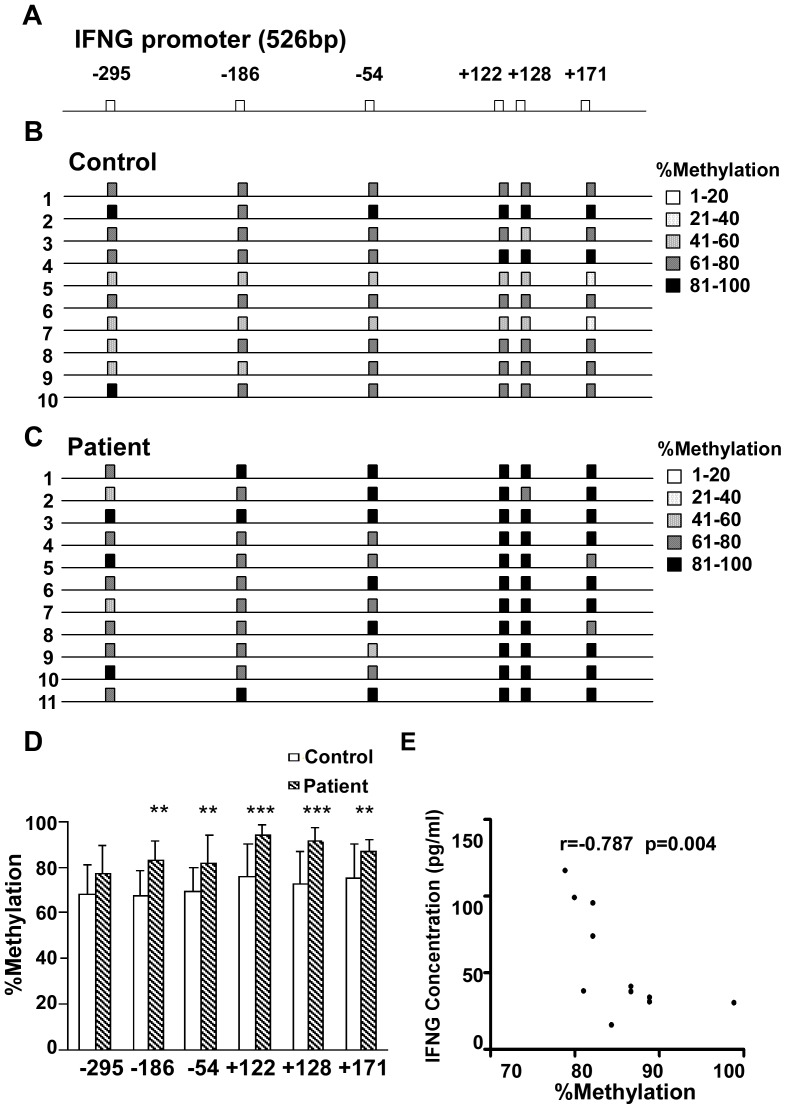
IFNG promoter methylation negatively correlated with IFNG levels. (**A**) A 526-bp region of the IFNG promoter was PCR-amplified from the bisulfite-treated reverse strand. Methylated cytosines resistant to bisulfite treatment were scored from 15 cloned PCR sequences for each individual and are denoted on the gene map as CpG (Squares). (**B**) Gene map of the methylated IFNG promoter in CD4^+^ T cells from ten healthy controls. The degree of methylation at each CpG site is depicted by the strength of shading. (**C**) Gene map of the methylated IFNG promoter in CD4^+^ T cells from 11 different lung cancer patients. The degree of methylation at each CpG site is depicted as described in B. (**D**) IFNG methylation of CpG sites in the IFNG promoter of CD4^+^ T cells was significantly different between lung cancer patients and healthy controls. Data shown are the percent methylation for each IFNG promoter CpG site. A Chi-square test contingency table was used to assign *P* values for lung cancer patients vs healthy controls. Comparisons after scoring the nominal variables for lung cancer patients/healthy controls and methylated/unmethylated in continuous data for each CpG site (error bars, SD, *, *P*<0.05; **, *P*<0.01, ***, *P*<0.001). (**E**) Correlation analysis for the total percent methylation with the IFNG plasma concentrations in lung cancer patients. The correlation was analyzed by Spearman’s coefficient.

CD4^+^ T cells were isolated from PBMCs of 11 patients and 10 controls. IFNG gene promoter regions 526 bp in length were examined by bisulfite sequencing PCR. 15 reverse strand sequences of each sample were scored for their methylation profile at CpG sites −295, −186, −54, +122, +128 and +171 relative to the transcription start site (**Fig. 2A**).

Bisulfite sequence of the IFNG promoter from lung cancer patients (n = 165 clones) revealed a significantly higher degree of methylation at CpG sites relative to those in healthy controls (n = 150 clones). Total methylation at CpG sites in lung cancer patients and healthy controls was 85.4% and 71.4%, respectively (*P*<0.001) (**Table 1**). We compared the percentage of specific CpG methylation sites for each sample. The IFNG promoter of CD4^+^ T cells displayed hypermethylation at 6 CpG sites in patients compared with healthy controls (**Fig. 2B, 2C**). In particular, CpG methylation at the IFNG promoter of patient CD4^+^ T cells was significantly higher at positions −186, −54, +122, +128 and +171 (p<0.01) by Pearson Chi-square test (**Fig. 2D** **)**. Among these positions, transcription factor binding sites occur at −186, −54, with sites at +122, +128 proximal to the transcription start site.

**Table 1:** Methylation at CpG sites of the IFNG promoter in CD4^+^ T cells of lung cancer patients and healthy controls.

Groups	Sequences	Methylated	Unmethylated
Lung cancer patients	165	845	145
Healthy controls	150	643	257***

CpG methylation (position −295, −186, −54, +122, +128, +171) was scored at specific sites in each IFNG promoter DNA sequence. The percentage of methylated CpG sites in lung cancer patients and healthy controls was 85.4%, and 71.4%, respectively. The number of methylated and unmethylated sites was scored and Chi-square analysis was used to assign p values (***, *P*<0.001).

To further evaluate the effect of methylation on IFNG expression, we calculated the correlation between plasma IFNG concentration and methylation rate in patients. Plasma IFNG concentrations were negatively correlated with the percent of IFNG promoter methylation in patient CD4^+^ T cells (r = −0.797, *P* = 0.004, **Fig. 2E**).

### Suppressed IFNG Expression of CD4^+^ T Cell in the SPC-A1 Transwell Culture System

**Figure 3:**
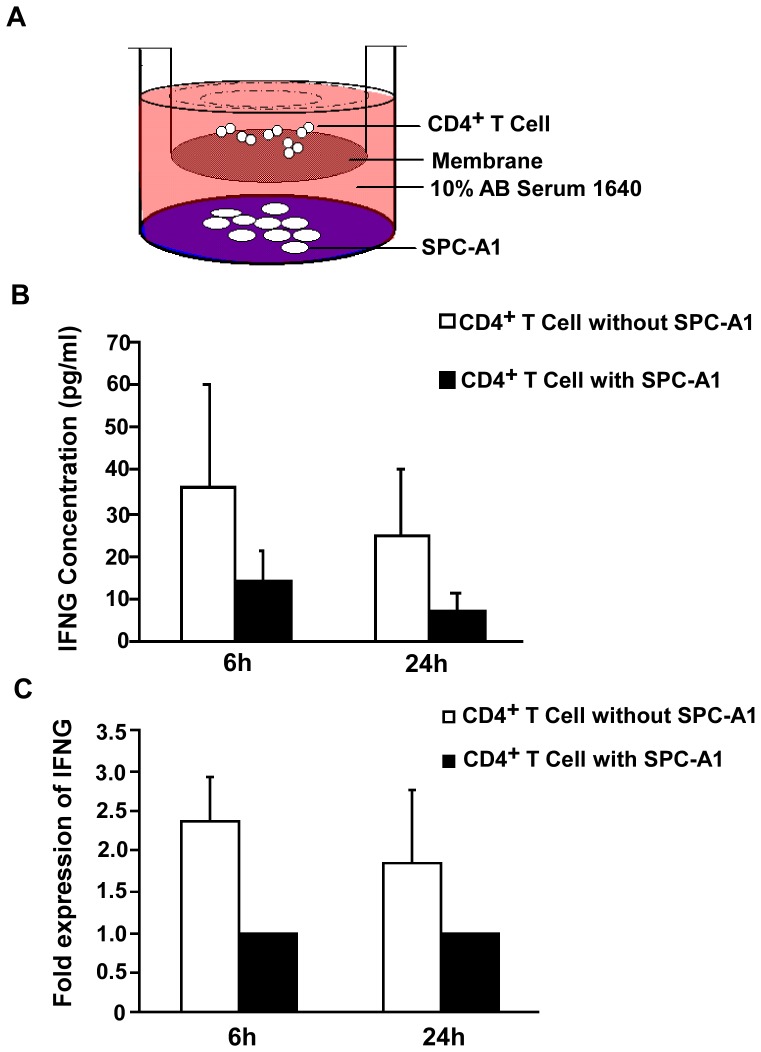
Suppressed IFNG expression of CD4^+^ T cell in the SPC-A1 transwell culture system. (**A**) CD4^+^ T cells co-cultured with SPC-A1. CD4^+^ T cells (6×10^5^ cells/well) and SPC-A1 (2×10^5^ cells/well) were grown separated by a 0.4 µm pore insert in 24-well culture plates. SPC-A1 cells were grown in the outer wells and CD4^+^ T cells were grown in suspension in the inner wells. (**B**) Production of IFNG in CD4^+^ T cells cultured with or without SPC-A1. Supernatants were collected after anti**-**CD3, or CD28 stimulation for 6 or 24 h, and IFNG detected by ELISA. Results are from experiments performed on CD4^+^ T cells from 6 healthy volunteers (error bars, SD). (**C**) Quantitative RT-PCR analysis of IFNG transcripts from CD4^+^ T cells cultured with or without SPC-A1. RNA was isolated after anti-CD3, or CD28 stimulation for 6 or 24 h. Results are from experiments performed on CD4^+^ T cells from 6 healthy volunteers. IFNG levels increased 2.37 fold when stimulated for 6 h in CD4^+^ T cells grown without SPC-A1 compared with CD4^+^ T cells grown with SPC-A1.

A transwell culturing system was used to investigate the effect of the lung cancer cell line SPC-A1 on IFNG expression of CD4^+^ T cells (**Fig. 3A**). CD4^+^ T cells isolated from the transwell culturing system with SPC-A1 showed poor IFNG production after anti-CD3 stimulation for 6 h or 24 h. In contrast, CD4^+^ T cells cultured in the absence of SPC-A1 showed a vigorous IFNG response after anti-CD3 stimulation for 6 h or 24 h. (*P<*0.05; *P<*0.05, **Fig. 3B**). Results of quantitative RT-PCR analysis also showed that CD4^+^ T cells cultured in the absence of SPC-A1 showed a vigorous IFNG mRNA expression after anti-CD3 stimulation for 6 h or 24 h, compared with CD4^+^ T cells co-cultured with SPC-A1 (*Fold = 2.37, P<*0.05; *Fold = 2.37, P<*0.05, **Fig. 3C**).

### Hypermethylation Status of the IFNG Promoter in CD4^+^ T Cells Co-cultured with SPC-A1

**Figure 4:**
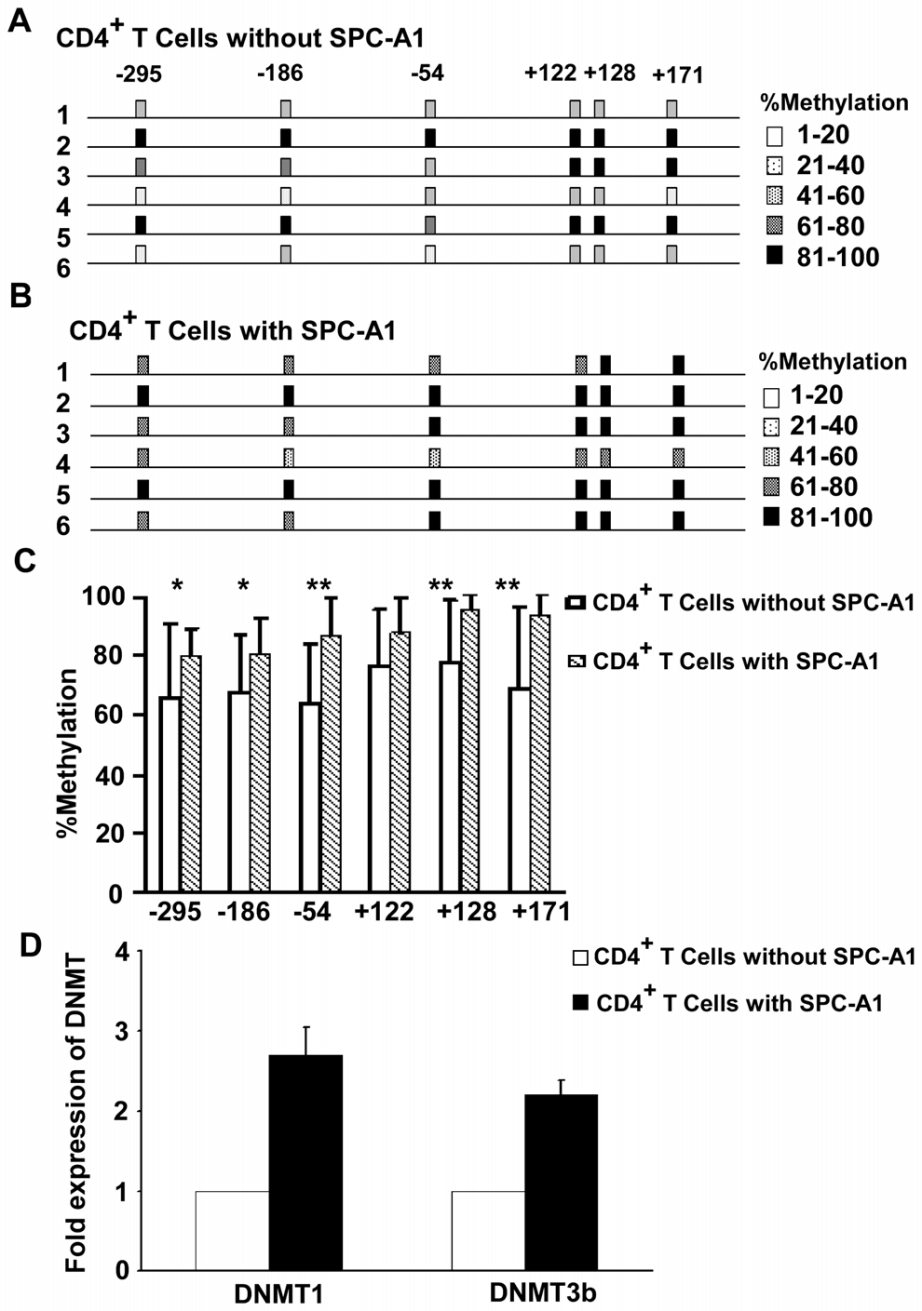
Hypermethylation status of the IFNG promoter in CD4^+^ T Cells co-cultured with SPC-A1. (**A**) Gene map of the methylated IFNG promoter in CD4^+^ T cells cultured without SPC-A1 from 6 healthy volunteers. The degree of methylation at each CpG site is depicted by the strength of shading. (**B**) Gene map of the hypermethylated IFNG promoter in CD4^+^ T cells cultured with SPC-A1 from 6 healthy volunteers. The degree of methylation at each CpG site is depicted by the strength of shading. (**C**) Methylation of CpG sites in the IFNG promoter was significantly different between CD4^+^ T cells cultured with or without SPC-A1 cells. Graph summarizing the percent of methylated CpG site observed at each position analyzed. IFNG promoter shows hypermethylation among CD4^+^ T cells cultured with SPC-A1 cells compared with CD4^+^ T cells cultured without SPC-A1 cells. Chi-square test contingency table was used to assign *P* values. Comparison of CD4^+^ T cells cultured with SPC-A1 cells vs CD4^+^ T cells cultured without SPC-A1 cells (error bars, SD, ^*^ *P*<0.05; ^**^ *P*<0.01). (**D**) Total RNA extraction and quantitative RT-PCR was performed to quantify DNMT1 and DNMT3b mRNA levels. The mRNA levels were normalized to β-actin. They were showed to be the ratios of CD4^+^ T cells cultured with or without SPC-A1 cells, from three independent experiments, expressed as mean ± SD.

The methylation status of the IFNG promoter in CD4^+^ T cells cultured with and without SPC-A1 cells was evaluated. The promoter displayed hypermethylation after co-cultured with SPC-A1 cells (n = 90 clones), showed a sharp contrast to the CD4^+^ T cells cultured without SPC-A1 cells (n = 90 clones). The percentage of CpG sites in CD4^+^ T cells cultured with or without SPC-A1 was 85.4% and 70.9%, respectively. The number of methylated and unmethylated sites was scored and Chi-square analysis was used to evaluate p values (***, *P*<0.001) (**Table 2**). We then compared the percent of specific CpG methylation sites for each group (**Fig. 4A, B**). The methylation percentage at the IFNG promoter was 80.0%–95.5% and 64.4%–78.9%, with and without SPC-A1 co-culture, respectively. The CpG sites at positions −295, −186, −54, +128 and +171, in particular, displayed significant differences (**Fig. 4C**).

**Table 2:** Methylation at CpG sites of the IFNG promoter in CD4^+^ T cells cultured with or without SPC-A1.

Groups	Sequences	Methylated	Unmethylated
CD4^+^ T Cells without SPC-A1	90	383	157
CD4^+^ T Cells with SPC-A1	90	461	79***

CpG methylation (position −295, −186, −54, +122, +128, +171) was scored at specific sites in the IFNG promoter for each DNA sequence. The percentage of methylated CpG sites in CD4^+^ T cells cultured with or without SPC-A1 was 85.4% and 70.9%, respectively. The number of methylated and unmethylated sites was scored and Chi-square analysis was used to assign p values (***, *P*<0.001).

DNMT1 and DNMT3b mRNA levels in CD4^+^ T cells cultured with SPC-A1 cells were up regulated compared with CD4^+^ T cells cultured alone (DNMT1 fold = 2.7, *P*<0.05; DNMT3b fold = 2.2, *P*<0.05).

## Discussion

A clearer picture of the dynamic relationship between the host immune system and tumor growth is emerging as the cells and molecules that participate in the natural anti-tumor immune response are being identified [11], [12]. Host immune system activation by IFNG is crucial for an anti-tumor response. Recent study showed that IFNG is vital to tumor surveillance by the immune system and that there was a high correlation between IFNG production and tumor regression during immunotherapy [5]. Mice lacking or deficient in IFNG receptors or STAT1 developed tumors more rapidly, and had a higher tumor frequency than wild type mice following a challenge with methylcholanthrene [13], [14].

It is well known that cytokine expression is controlled via a network of transcription factors and epigenetic modifications [15]–[18]. Human studies have shown that IFNG production in adult peripheral blood and cord blood is regulated by CpG methylation at sites within or adjacent to the IFNG promoter in CD4^+^/CD45RO^−^ T cells [19], [20]. In addition, a role for IFNG promoter methylation in atopic syndrome has been suggested [21], which pointed to methylation as a mechanism for controlling expression. Bronchial asthma patients display hypermethylation of the IFNG gene in CD4^+^ T cells following allergen challenge which correlated with decreased IFNG expression [22], [23]. Similar findings have also been reported in PBMCs from acute-on-chronic hepatitis B liver failure and colon cancer patients [24], [25]. All of these observations suggest DNA methylation is an important epigenetic mechanism that involves in IFNG expression regulation.

In our study, we observed decreased levels of plasma IFNG and intracellular IFNG in CD4^+^ T cells from lung cancer patients. These results suggest that IFNG expressed lower in lung cancer. An accumulating body of evidence has implicated CpG methylation of the IFNG promoter as an important negative transcriptional regulator of IFNG production in human T cells [26], [27]. In the present study, we applied bisulfite sequencing to determine the CpG methylation status of the IFNG promoter. A significant negative correlation between IFNG plasma levels and total IFNG promoter methylation in CD4^+^ T cells of lung cancer patients was observed. To the best of our knowledge, this is the first study to suggest that IFNG promoter methylation may influence IFNG expression in lung cancer. Our results are consistent with findings that the epigenetic methylation status of IFNG may play a crucial role in modulating mucosal cytokine secretion [28] and in bronchial asthma patients [22].

We also compared the methylation status of several CpG sites. In lung cancer patients, five of six CpG sites (−295, −186, −54, +122, +128, +171) analyzed were significantly hypermethylated (the only exception was site −295). In particular, the degree of methylation increased prominently at +122, and +128, suggesting a close relationship with IFNG expression. We found a prominent increase in the degree of methylation at positions −295, −186, −54, +128 and +171 (without changing at site +122) in CD4^+^ T cells co-cultured with the lung adenocarcinoma cell line SPC-A1. In particular, the degree of methylation increased prominently at positions −54, +128, and +171. This result showed a little difference from the result observed in lung cancer patients, as the existence of environment variance between in vitro and in vivo condition.

There is great interest in better understanding the molecular mechanisms leading to altered methylation of the IFNG gene promoter during tumorigenesis [29], [30]. Until recently, there was little data on whether lung cancer cells act directly on immune cells to induce hypermethylation of the IFNG promoter. In our research, the hypermethylation status in CD4^+^ T cells in lung cancer patients suggests that the presence of cancerous cells may contribute to an altered expression profile.

Microenvironments play a critical role in tumor progression where immune-resistant tumor variants are selected [31]. Tumor-derived soluble factors impel various mechanisms to escape immune attack in the tumor microenvironment [32], [33]. In the transwell culturing system applied here, we found clear differences in the epigenetic regulation of the IFNG promoter between CD4^+^ T cells from healthy individuals cultured with and without SPC-A1. The result of IFNG promoter methylation of CpG sites demonstrated hypermethylation was similar to that observed in CD4^+^ T cells of lung cancer patients (85.4% vs 85.4%), which generated lower levels of IFNG upon activation. Interestingly, Janson et al had reported that tumor-infiltrating CD4^+^ T cells were inappropriately hypermethylated in colon cancer patients [34].

SPC-A1 cells could act on CD4^+^ T cells of healthy volunteers to induce hypermethylation without direct contact, suggesting the presence of a soluble, noncontact dependent micro-factor(s). Studies have shown that DNA methylation is catalyzed by DNMTs, including the maintenance methyltransferase DNMT1 that acts on hemi-methylated substrates to maintain methylation patterns after DNA replication [35], [36]. In addition, there are the de novo methyltransferases DNMT3a and DNMT3b that catalyze methylation of unmethylated DNA [37], [38]. Alterations in DNMT expression correlate with changes in genomic DNA methylation, and are well described in many cancers [39]–[44]. Upon examination of DNMT expression patterns in our model, we observed a marked increase in DNMT1 and DNMT3b mRNA expression in CD4^+^ T cells cultured with SPC-A1 cells. Therefore, we speculated that soluble, noncontact dependent factors may induce hypermethylation of the IFNG promoter by stimulating DNA methyltransferase activity, which then downregulate IFNG secretion. These factors may be cytokines, nucleic acids, or microRNAs secreted or derived by lung cancer cells [45]–[47]. However, additional research will be needed confirm this hypothesis. These in vitro results suggest that promoter methylation may influence IFNG expression in lung cancer patients. Methylation-mediated IFNG decreases may benefit tumor survival and shift the balance of tumor immunity towards tumor progression.

We have previously shown that decreased expression of tumor suppressor genes is associated with aberrant CpG island methylation in gene promoter regions in lung cancer patients, and that expression could be restored by demethylation [48]. This suggested that demethylation as mediated by 5-aza-dC might serve as a useful approach for treating lung cancer [48]–[50]. Therefore, treatment of lung cancer through demethylation, which increases expression not only of tumor suppressor genes, but also of cytokine genes such as IFNG, help protect against tumors.

In conclusion, IFNG promoter methylation is associated with decreased IFNG expression in lung cancer patients. The interaction between lung cancer cells and CD4^+^ T cells induces hypermethylation of the IFNG promoter in CD4^+^ T cells, which serve as a mechanism of tumor-induced immunosuppression.

## Materials and Methods

### Ethics Statement

This study was approved by the Committee on the Ethics of Treatment of Human Subjects of the First Affiliated Hospital of Nanjing Medical University (Permit Number: 20A6-2869), and a written informed consent was also obtained from each participant.

### Selection of Lung Cancer Patients and Healthy Controls

Lung cancer patients (N = 30) and healthy adults (N = 30) were from the first affiliated hospital of Nanjing Medical University (Nanjing, China). Lung cancer patients were diagnosed as carcinoma by pathologic diagnosis. Patients who received preoperative chemotherapy, radiotherapy or operation before collecting blood sample had been excluded. The detailed clinical data (including age, gender, smoking history) were obtained from each object’s medical records and listed in **Table 3**. Among lung cancer patients, there were 24 cases of adenocarcinoma, 2 cases of squamous carcinoma, 2 cases of alveolar cell carcinoma, and 2 cases of poorly differentiated carcinoma.

**Table 3:** Demographic data for lung cancer patients and healthy controls in the current study.

Clinical pathologic characteristics	Lung adenocarcinoma (n = 30)	Healthy controls (n = 30)
Age (years)		
<58	18	18
≥58	12	12
Gender		
Male	22	22
Female	8	8
Smoking		
Never	11	12
Ever	19	18

### Blood Sample Collection and Processing

Venous blood (10 ml) with EDTA-K_2_ was collected from healthy controls and lung cancer patients before operation. Plasma was separated and stored at −70°C prior to measuring IFNG. Peripheral blood mononuclear cells (PBMCs) were separated by Ficoll-Hypaque density gradient centrifugation (GE Health Care Life Sciences, Piscataway, NJ, USA). CD4^+^ T cells were isolated from PBMCs using a CD4-positive isolation kit (Dynal, Oslo, Norway). The purity of isolated CD4^+^ T cells was determined by flow cytometry using PE-conjugated anti-CD4 (Beckman, Marseille, France). CD4^+^ T cells were then collected for DNA extraction, bisulfite modification, and sequencing.

### Cell Lines and Culture Conditions

The human NSCLC cell line SPC-A1 was purchased from the Chinese Academy of Sciences in Shanghai, and cultured in 5% CO_2_ in RPMI 1640 with 2 mmol/L L-glutamine, 10% fetal bovine serum (Invitrogen, Carlsbad, CA, USA), penicillin G (50 U/mL), and streptomycin (50 µg/mL).

### CD4^+^ T Cells Co-cultured in the Transwell Culturing System

Transwell experiments were performed in 24 well plates with pore size 0.4 µm (Corning Costar, Corning, NY, USA). SPC-A1 cells (2×10^5^) were cultured in the outer wells of 24 well plates in 1640-medium supplemented with 10% human AB serum. CD4^+^ T cells (6×10^5^) separated from healthy adults were added into the inner wells in the same medium. As shown in Fig. 3, control groups were established with CD4^+^ T cells growing in the inner wells without SPC-A1 cells. After 5 days of culture, CD4^+^ T cells were washed, and 1×10^6^ cells were collected for DNA extraction, while the remaining was transferred to 96-well culture plates. 5×10^4^ cells/well were stimulated with 1 µg/ml soluble anti-CD3 (eBioscience, San Diego, CA, USA) and 1 µg/ml soluble anti-CD28 (eBioscience) (in a total volume of 200 µl) for 6 or 24 h. After stimulating, supernatants were collected for IFNG detection by ELISA, CD4^+^ T cells were collected for IFNG mRNA analysis.

### ELISA

IFNG levels in culture supernatants and plasma were determined by ELISA (R&D Systems, Minneapolis, MN, USA), according to the manufacturer’s protocol.

### FACS Analysis

PBMCs were stimulated with PMA (10 ng/mL). Brefeldin A (Sigma-Aldrich, St. Louis, MO, USA) was added at a final concentration of 10 µg/ml and incubated 4 h. PBMCs samples were taken from responding cultures and stained with a combination of fluorochrome-conjugated mAbs consisting of anti**-**CD8-PE-CY5 and anti**-**CD3-FITC (BD PharMingen, San Diego, CA, USA). After fixation and permeabilization (Fix Perm, Caltag, Buckingham, UK), cells were stained with anti-IFNG-PE and isotypic mAbs (BD PharMingen). Isotype-matched control antibodies were used to determine the level of background staining for the mAb mixture. All events were acquired using a FACSCalibur flow cytometer (BD Biosciences, San Jose, CA, USA). Data were analyzed with CELL Quest software (BD PharMingen).

### RNA Isolation and Quantitative Real-time PCR

RNA isolation was performed using miRNeasy Mini Kit (Qiagen, Germantown, MD, USA) and reverse transcribed using a PrimeScript RT Reagent Kit (TaKaRa, Tokyo, Japan) according to the manufacturer’s protocol. cDNA was analyzed for the expression of target genes and quantified by RT-PCR using SYBR Premix Ex Taq™.Quantitative PCR was performed on an ABI 7500 real-time PCR system (Applied Biosystems, Foster City, CA, USA). The PCR primers used were: IFN-γ-forward, 5′-GCA GGT CAT TCA GAT GTA GCG G-3′; IFN-γ-reverse, 5′-TGT CTT CCT TGA TGG TCT CCA CAC-3′; DNMT1-forward, 5′-GAT CGA ATT CAT GCC GGC GCG TAC CGC CCC AG-3′; DNMT1-reverse, 5′-ATG GTG GTT TGC CTG GTG C-3′; DNMT3b-forward, 5′-CCT GCT GAA TTA CTC ACG CCC C-3′; DNMT3b-reverse, 5′-GTC TGT GTA GTG CAC AGG AAA GCC-3′; β-actin-forward, 5′-TGG CAC CCA GCA CAA TGA A-3′; β-actin-reverse, 5′-CTA AGT CAT AGT CCG CCT AGA AGC A-3′. Ct values were normalized to expression in CD4^+^ T cells using the 2^−ΔΔCt^ method and β-actin as the housekeeping gene. Experiments were done in triplicate, and results from three independent experiments were averaged.

### DNA Isolation and Methylation Analysis

Methylation analysis was conducted using bisulfite sequencing. Genomic DNA of CD4^+^ T cells was isolated using QIAamp Mini Kit (Qiagen) as recommended by the manufacturer. The DNA was bisulfite converted using CpGenome™ DNA modification Kit (Millipore Corporation, Billerica, MA, USA) according to the manufacture’s instructions. The primers used to amplify the IFNG promoter were: IFNG-forward, 5′-TGT GAA TGA AGA GTT AAT ATT TTA TTA-3′; IFNG-reverse, 5′-TTG GTA GTA ATA GTT AAG AGA ATT TA-3′ [19]. PCR products were generated using the bisulfite-treated DNA as template.

The most prominent PCR band was separated on 2% agarose gel electrophoresis, and purified by gel extraction (Qiagen), treated with TA cloning using DNA-Tailing (TaKaRa), and sequenced on an ABI 3730 (Applied Biosystems). All operations were performed by GeneScript Corporation (a sino-America joint venture, Nanjing, China). Sequences were analyzed by bioinformatics software Chromas Version 1.45 (Technelysium, South Brisbane, Australia).

### Statistical Analysis

All data were analyzed using SPSS 16.0 software (IBM, Chicago, IL, USA). Independent t tests were used to compare plasma IFNG levels and intracellular differences between patients and healthy controls. The data are summarized as mean±SD. Methylation differences in the IFNG promoter between different groups were assessed using a Pearson Chi-square test. *P* values less than 0.05 were considered statistically significant.
